# Psychometric Properties of the Independent 36-Item PID5BF+M for ICD-11 in the Czech-Speaking Community Sample

**DOI:** 10.3389/fpsyt.2021.643270

**Published:** 2021-05-26

**Authors:** Karel D. Riegel, Albert J. Ksinan, Lucia Schlosserova

**Affiliations:** ^1^Department of Addictology, General University Hospital in Prague and 1st Faculty of Medicine, Charles University, Prague, Czechia; ^2^Research Centre for Toxic Compounds in the Environment (RECETOX), Faculty of Science, Masaryk University, Brno, Czechia

**Keywords:** ICD-11, DSM-5 AMPD, personality disorder classification, PID5BF+M, trait diagnosis, borderline pattern qualifier

## Abstract

**Background:** Empirical soundness and international robustness of the PID5BF+M, a shortened version of the PID-5 developed for simultaneous evaluation of maladaptive personality traits in the DSM-5 AMPD and ICD-11 models for personality disorders, was recently confirmed in 16 samples from different countries. Because the modified PID5BF+ scale (36 items) was extracted from the complete 220-item PID-5, an independent evaluation of psychometric properties of a stand-alone PID5BF+M is still missing.

**Objectives:** The present study evaluated the validity and reliability of the 36-item PID5BF+M in comparison with the extracted version from the original PID-5. It also assessed associations between the Borderline Pattern qualifier and trait domain qualifiers.

**Methods:** Two non-clinical samples meeting the inclusion criteria were employed in the study. Sample 1 (*n* = 614) completed the 220-item PID-5; Sample 2 (*n* = 1,040) completed the independent 36-item PID5BF+M. Participants were from all 14 regions of the Czech Republic. The Borderline Pattern qualifier was evaluated using a shortened IPDEQ screener.

**Results:** The proposed latent structure of the independent PID5BF+M was confirmed, with an exception of the Disinhibition domain. The results confirmed good internal consistency and test-retest reliability of the measure, as well as some support for the measurement invariance of the independent PID5BF+M in comparison with the extracted version from the original PID-5. Significant associations between the Negative affectivity, Disinhibition, and Psychoticism qualifiers and the IPDEQ items for the emotionally unstable personality disorder of both impulsive and borderline types confirmed good predictive validity of the PID5BF+M in pursuing borderline psychopathology within the ICD-11 model.

**Conclusions:** The independent PID5BF+M was found to be a valid and reliable tool for evaluation of the ICD-11 trait model. However, the Disinhibition domain deserves further investigation in clinical samples as well as in international community samples.

## Introduction

Both the Model for Personality Disorders (PDs) in the 11th edition of the International Classification of Diseases (ICD-11) and the Alternative DSM-5 Model for Personality Disorders (AMPD) use personality trait domains to specify individual manifestations of personality psychopathology beyond the evaluation of the overall personality impairment severity (1). The trait qualifiers in ICD-11 not only offer empirically-informed and homogeneous basis for personality psychopathology convergent with other empirically derived dimensional models (2) and AMPD (3), but they also contribute clinical information necessary for the selection of the type and the focus of psychotherapy (4). In addition, the significant overlap between ICD-11 and the AMPD dimensional models of personality traits allows the instruments originally developed for AMPD, namely the Personality Inventory for the DSM-5 (PID-5) (5), to be used for the operationalization of both models. So far, the original 220-item PID-5 has served as a methodological basis from which several shortened versions were derived (6–8). At the same time, the PID-5 was used to assess the criterion validity of the newly emerging measures for assessing the personality trait qualifiers in ICD-11 (9, 10).

Although both ICD-11 and AMPD include Negative affectivity, Detachment, Antagonism/Dissociality, and Disinhibition among the five domains of personality traits, some differences can be noted between these models that make it impossible for a clinician to switch seamlessly between the two nomenclatures when describing a patient. In contrast to AMPD, the main differences in ICD-11 include the omission of personality traits qualifiers in relation to schizotypy and psychoticism; the inclusion of the Anankastia qualifier; and the absence of specific trait facets delineating individual qualifiers (11). Another difference between the two models is the preservation of the ICD-10 criteria for emotionally unstable PD in the form of a Borderline Pattern qualifier in ICD-11 (12). This step reflects that research studies into borderline PD have far outnumbered those for other categorical diagnoses (13). From the clinical perspective, it can be used to explain behaviors such as self-harm, to exclude patients from the standard treatments for other diagnoses, to offer treatments for the condition itself, and to recruit patients to research trials or services because it is so prevalent, and it has a sufficiently robust intervention base to allow insurance companies to issue contracts for treatment (14). Generally speaking, there is an evidence that many clinical professionals in many respects prefer the AMPD dimensional model of personality traits to the current categorical approach to PDs (15). Synchronization of both dimensional conceptualizations would therefore seem to be a meaningful step in the further development of the ICD-11 model, which would reflect the needs of both the mental health professionals in terms of clinical applicability and researchers in terms of empirical validity and comparability with AMPD.

In an effort to synchronize these two models, an algorithm has recently been developed to evaluate the combined AMPD and ICD-11 personality traits model based on six higher-order domains (i.e., Negative affectivity, Detachment, Antagonism, Disinhibition, Anankastia and Psychoticism), covering 17 of the lower-order facets, and featuring a total number of 34 items. This algorithm is captured by the Personality Inventory for DSM-5 - Brief Form Plus (PID-5BF+) (16). Authors applied Ant colony optimization algorithms to select a set of items that maximizes the reliability and validity of the trait domain and facet scales while providing a good model fit of the measurement model as well as cross-cultural measurement invariance. While latent structure, reliability, and criterion validity were ascertained in three different German- and English-speaking samples and in two separate German-speaking validation samples and the measure was able to discriminate personality disorders from other diagnoses in a clinical subsample, results suggested further modifications for capturing ICD-11 Anankastia. The operationalization of the Anankastia domain in PID-5BF+ based on the PID-5 facets of Rigid Perfectionism and Perseveration is consistent with an empirically derived crosswalk between the AMPD and ICD-11 personality trait domains (3, 4). However, this approach does not capture the anankastic features (i.e., the features resembling, among other things, the conceptualization of the anal character in the traditional psychoanalytic thinking) in their clinical entirety, as it omits the feature of Orderliness (17). For this reason, a modified 36-item version of the PID-5BF+ (PID5BF+M) was developed. Consistently with the initial 37-facet version of the DSM-5 trait model (5), Bach et al. (11) extracted subfacets of orderliness, rigidity, and perfectionism from the composite facet of rigid perfectionism in order to further adapt the PID5BF+ to efficiently capture the primary facets represented in the ICD-11 domain of Anankastia. Moreover, in this modified version of PID5BF+ authors omitted perseveration as a primary feature of Anankastia because this facet was originally intended to capture features of Negative affectivity as reflected by its expected loadings on the Negative affectivity domain (18). Recent findings generally supported the empirical soundness and international robustness of the 6 PID5BF+M domains across 16 samples from different countries, regions, and populations, as well as meaningful associations with familiar interview-rated PD types (11). Nevertheless, these datasets were extracted from the original 220-item version of PID-5. Bearing in mind, the similarity of correlations of the original PID-5 scales and the PID5BF+M scales with other measures are likely to be inflated when the PID5BF+M scales are derived from the original PID-5 (19). For this reason, it is important to examine the PID5BF+M as a standalone measure as compared to extracting its items from the original PID-5.

The aim of this study was to verify the psychometric properties of the independent 36-item version of PID5BF+M and to compare them with the extracted PID5BF+M version from the original PID-5 by testing them for invariance. Given that the focus of the study was to primarily verify the general factor structure of the independent version of the measure, a thorough analysis of the validity of the separate Anankastia domain was not among the main aims of this study. At the same time, we examined the associations between the Borderline Pattern qualifier and the qualifiers of personality traits with respect to the proven continuity of PID5BF+M with specific diagnoses of PDs (11). We hypothesize that there would be a substantial relationship between the ICD-10 criteria for emotionally unstable PD based on self-assessment and the PID5BF+M domains of Negative affectivity and Disinhibition. This hypothesis is in line with the proposed trait associations for borderline PD in the DSM-5 as well as with empirical findings on the association of PID-5 traits and borderline PD (16, 20).

## Methods

### Samples and Procedures

Two samples of volunteers from the general population were used. They consisted of university students from various fields of study, working volunteers and pensioners. To be included in the study, volunteers in both groups needed to fulfill the criterion of being ≥ 18 years of age. Participation in the study was voluntary and anonymous for all respondents, and all participants were asked to give their informed consent to participate in the study, which they had the opportunity to withdraw at any time without stating the reason. Participants were not rewarded for their participation in the study; however, if they were interested in feedback, they could provide us with their email address. The ethics committee of the General university Hospital in Prague approved the study protocol and the informed consent form.

After removing participants based on their PID-5 Response Inconsistency Scale (PID-5-RIS) score (*n* = 12; see Plan of analysis), Sample 1 (*n* = 614) was used to compare the psychometric properties of PID5BF+M extracted from the 220-item PID-5 with the Sample 2, in which the independent PID5BF+M was administered. The group consisted of respondents included in the international study by Bach et al. (11) (*n* = 372), extended by a subgroup of candidates applying to join the Police of the Czech Republic (*n* = 254). Gender representation was balanced: there were slightly more women (*n* = 313, 51.0%) than men (*n* = 301, 49.0%). Age range was 18–84 years (*M* = 30.63, *SD* = 11.09). Distribution according to the highest attained level of education in this group was as follows: primary education 0.9%; secondary education 56.5%, some college 4.0%, undergraduate degree 17.6%, graduate degree 21.0%.

After removing individuals with patterned responses (*n* = 22, see Plan of analysis) Sample 2 included *n* = 1,040 individuals. Gender representation was unbalanced, as there were more women (*n* = 700, 67.3%) than men (*n* = 340, 32.7%). Age range was 18–87 years (*M* = 35.15, *SD* = 12.18). Distribution according to the highest attained level of education in the group was as follows: primary education 2.0%; secondary education 36.5%; some college 3.6%; undergraduate degree 18.2%; graduate degree 39.7%. The group consisted of respondents from all 14 regions of the Czech Republic, however, the representation was not uniform (min/max number of respondents per region = 18/224). Overall, 16.3% of respondents (*n* = 169) reported experience with psychiatric treatment in the past. This approximately corresponds to the estimated 21.9% prevalence of various mental disorders in the general Czech population (21).

Questionnaires were administered to Sample 2 individually, to be filled either by the paper-and-pencil method or online. In the case of paper-and-pencil administration, respondents were asked to carefully read the instructions before starting the questionnaire. Trained administrators were present during the administration to respond to the possible technical queries of respondents. Online data collection was limited to 3 months. During this period, respondents were addressed anonymously through adverts on social media and relevant websites. They would complete the questionnaires upon accessing a link provided in the advert and were asked to answer all items. Some respondents (*n* = 201) were asked to fill in the questionnaire twice to verify the test-retest reliability. The time interval between the first and second administration ranged from 1 to 15 weeks (*M* = 46.5 days, median = 42 days, min/max = 8/102 days). Data collection for Sample 1 was similar, for more detailed information please refer to the studies by Riegel et al. (8, 22). The Police applicants subgroup completed the questionnaires individually by the paper-and-pencil method.

Given that Sample 1 and Sample 2 were drafted from different populations, they showed differences when compared on the demographic characteristics. Specifically, the samples differed on sex, as the proportion of females was higher in Sample 2 than in Sample 1 (67 vs. 51%), $\chi_{\left(1\right)}^{2}$ = 42.69, *p* < 0.001. Sample 2 was also older on average (*M* = 35.15 years vs. 30.63 years), *t*_(1383.6)_ = 7.80, *p* < 0.001. Finally, Sample 2 had higher level of attained education, as Sample 1 was predominantly high school graduates (81%) given that it included the subgroup of Police applicants, $\chi_{\left(4\right)}^{2}$ = 324.90, *p* < 0.001.

### Instruments

We used the self-report PID5BF+M to operationalize the ICD-11 and DSM-5 domains of personality traits. The complete PID5BF+M consists of 18 facets assessed through 36 items (2 items per facet), rated on a 4-point Likert scale (0 = very untrue or often untrue; 1 = sometimes or somewhat untrue; 2 = sometimes or somewhat true; and 3 = very true or often true). The 6 domains have been calculated based on the average scores of the three primary facets of each particular domain: Negative affectivity has been calculated from the average scores of the facets of emotional lability, anxiousness, separation insecurity; Detachment from the facets of withdrawal, anhedonia, intimacy avoidance; Antagonism from the facets of manipulativeness, deceitfulness, grandiosity; Disinhibition from the facets of irresponsibility, impulsivity, distractibility; Psychoticism from the facets of unusual beliefs and experiences, eccentricity, perceptual dysregulation; and Anankastia from the facets of perfectionism, rigidity, and orderliness. An independent version of the PID5BF+M was administered to Sample 2, while Sample 1 assessment included the PID5BF+M extracted from the 220-item version of PID-5, in accordance with previous studies (11, 16). For more information on the translation and validation of the Czech version of PID-5, please refer to the relevant studies (8, 22).

We administered selected items of the self-reported International Personality Disorder Examination Questionnaire (IPDEQ) (23) in Sample 2 to assess the associations between the Borderline Pattern qualifier and other PID5BF+M personality trait qualifiers. IPDEQ is a screener for ICD-10 PDs consisting of 59 yes/no items. For the purpose of this study we employed 10 items related to the ICD-10 criteria for emotionally unstable PD and, in accordance with the ICD-11 Borderline Pattern qualifier (4), corresponding to the features of borderline psychopathology.

### Plan of Analysis

To ensure the validity of data in Sample 1, we used the PID-5-RIS developed by Keeley et al. (24), which has proven successful in detecting random responses in the original version of PID-5 and has been verified by a number of recent studies (25–27). In line with these studies, we excluded respondents with a PID-5-RIS score ≥ 17. In Sample 2, we excluded respondents who answered 90% or more of the PID5BF+M items with the same value, i.e., the same value in >32 items.

As the first step, we estimated the fit of the six-factor model in Sample 1, which was administered the full 220-item version of the PID-5, using exploratory structural equation modeling (ESEM). This was first estimated in a multigroup model to evaluate whether there were any differences in the subgroups, i.e., the Police applicants and the community sample. As no substantial differences were found, we merged the subgroups into a single group to increase the sample size. Subsequently, the model was re-evaluated in terms of model fit, and the pattern of factor loadings.

In the next step, we fit the model in Sample 2, which was administered the independent 36-item PID5BF+M. Again, this was first assessed as a multigroup model to evaluate the differences in the methods of administration (paper-pencil vs. online). These methods have been previously found to be invariant in Sample 1 (22). As no differences were found across the modes of administration, the subgroups were merged, and the model fit and factor loadings were examined. We also tested the independent PID5BF+M in a five-domain model, excluding Psychoticism to be consistent with ICD-11 trait domain qualifiers.

Subsequently, we estimated the internal consistency of each facet using polychoric correlations (as there are only two items per facet) and McDonald's omega for the reliability of the domain scores. We also examined the test-retest reliability in Sample 2. The convergent validity of the shortened 36-item version extracted from the full version was assessed by comparing correlations of 15 facets and 5 domains defined by the 220-item version in Sample 1 (we omitted the Anankastia domain and its relevant facets of perfectionism, rigidity, and orderliness, which was not part of the original PID-5).

Moreover, we directly compared the model fit of the extracted and the independent version PID5BF+M by combining Sample 1 and 2 in a multigroup model and testing whether there was support for measurement invariance. Finally, the predictive validity of the independent 36-item PID5BF+M was tested by examining the associations of the six hypothesized domains and the two types of emotionally unstable PDs (i.e., borderline and impulsive) indexed by the IPDEQ, as well as of the emotionally unstable PD as a whole.

All ESEM models were estimated in Mplus 8 (28) with GEOMIN rotation and maximum likelihood with robust standard errors (MLR) as the estimator. To compare nested models (for measurement invariance testing), we used a Satorra-Bentler corrected chi-square difference test, as well as relative differences in additional fit indices, as chi-square difference testing is known to be affected by larger sample size so that it is more likely to be significant in larger samples (29). For absolute model fit, the cut-off values of CFI > 0.90 and RMSEA <0.08 were considered, as well as 90% confidence intervals of RMSEA. According to Cheung and Rensvold (30), nested models can be considered invariant if the difference in CFI is <0.01, and <0.01 in RMSEA.

## Results

### Model Fit and Latent Structure

First, we compared the model fit of the community and the Police applicants subsamples, comprising Sample 1. The six-factor ESEM extracted from the original 220-item PID-5 was estimated in a multigroup model within each group. There were minimal differences between the configural (loadings and intercepts freely estimated in each group) and the scalar (loadings and intercepts fixed to equality across groups) model, S-B Δ$\chi_{\left(84\right)}^{2}$ = 113.68, *p* = 0.017, ΔCFI = −0.011, ΔRMSEA = < −0.0001. This suggests that the model fit is not substantially different across the community and the Police applicants subsamples. For this reason, we decided to merge the groups to increase the sample size and the statistical power. The fit of the 6-factor ESEM model in Sample 1 was good, $\chi_{\left(60\right)}^{2}$ = 80.84, *p* = 0.038, CFI = 0.992, RMSEA = 0.024, 90% RMSEA CI [0.01, 0.04]. The pattern of standardized loadings of the 18 facets is shown in Table 1. All the facets showed the highest loadings on their respective factors, indicating that the six domains are mostly well-defined by three facets each. There were two exceptions: irresponsibility, which showed the highest loading (λ = 0.37) on the Psychoticism domain instead of its proposed primary domain of Disinhibition (λ = 0.36); and separation insecurity, which showed the highest loading (λ = 0.33) on Disinhibition instead of its proposed primary domain of Negative affectivity (λ = 0.31).

**Table 1:** Loadings patterns of the facets derived from the extracted version of PID5BF+M.

	**Negative affectivity**	**Detachment**	**Antagonism**	**Disinhibition**	**Anankastia**	**Psychoticism**
Emotional lability	**0.44**	−0.04	0.01	0.29	0.06	0.14
Anxiousness	**0.88**	0.11	0.03	−0.04	0.00	0.03
Separation insecurity	**0.31**	0.00	0.06	*0.33*	0.16	−0.06
Withdrawal	0.12	**0.70**	0.05	−0.12	−0.01	0.08
Anhedonia	0.09	**0.52**	0.09	0.27	0.04	−0.10
Intimacy avoidance	−0.07	**0.42**	0.04	0.05	0.20	0.07
Manipulativeness	0.00	0.09	**0.83**	−0.04	−0.05	0.07
Deceitfulness	0.10	0.01	**0.55**	0.22	0.10	0.02
Grandiosity	0.12	0.08	**0.38**	−0.01	0.18	0.17
Irresponsibility	−0.06	0.14	0.23	**0.36**	−0.11	*0.37*
Impulsivity	0.05	0.03	0.07	**0.69**	0.01	0.07
Distractibility	0.25	0.21	−0.07	**0.36**	−0.10	0.27
Perfectionism	−0.01	0.03	−0.01	0.04	**0.74**	0.05
Rigidity	0.02	0.09	0.07	−0.10	**0.53**	0.03
Orderliness	0.19	0.06	0.03	0.02	**0.53**	0.02
Unusual beliefs and experiences	0.10	0.05	0.17	−0.01	0.05	**0.56**
Eccentricity	0.08	0.07	0.08	0.04	0.07	**0.67**
Perceptual dysregulation	0.06	0.05	0.09	0.21	0.15	**0.29**

*Bolded are expected primary loadings of the facets (all of their loadings significant at p < 0.001). Italicized are non-primary loadings > |0.30|*.

In the next step, we tested the same model for individuals who were administered the independent 36-item PID5BF+M. First, we tested whether the type of administration (paper-and-pencil vs. online) affected the results in a multigroup model. The results for the multigroup model showed a minimal difference between the configural and the scalar model, S-B Δ$\chi_{\left(84\right)}^{2}$ = 117.91, *p* = 0.009, ΔCFI = −0.004, ΔRMSEA = 0.009, suggesting that the type of administration did not substantially alter the model structure, facet loadings, or item intercepts. In the next step, we estimated the fit of the model using the full sample. The fit of the 6-factor ESEM model in this group was good, $\chi_{\left(60\right)}^{2}$ = 179.96, *p* < 0.001, CFI = 0.971, RMSEA = 0.044, 90% RMSEA CI [0.036, 0.051]. The pattern of standardized loadings of the 18 facets is shown in Table 2. Five out of six domains, namely Negative affectivity, Detachment, Antagonism, Anankastia and Psychoticism showed expected patterns of loadings. However, the Disinhibition domain was not well-defined, as the loadings of the respective facets (i.e., irresponsibility, impulsivity, distractibility) were rather low and, in the case of the irresponsibility facet, not statistically significant (*p* = 0.053). Instead, this domain was better defined by the separation insecurity facet (λ = 0.56), followed by substantial loadings by anhedonia, grandiosity, anxiousness. and orderliness^1^. Interestingly, separation insecurity loaded primarily on Disinhibition and only secondarily on Negative affectivity.

**Table 2:** Loadings patterns of the facets derived from the independent version of PID5BF+M.

	**Negative affectivity**	**Detachment**	**Antagonism**	Disinhibition^*^	**Anankastia**	**Psychoticism**
Emotional lability	**0.80**	0.01	0.01	0.02	0.07	0.05
Anxiousness	**0.38**	0.29	−0.05	0.28	0.07	−0.02
Separation insecurity	**0.23**	−0.14	0.07	*0.56*	0.04	−0.07
Withdrawal	0.05	**0.77**	0.04	−0.13	0.07	0.01
Anhedonia	−0.08	**0.43**	0.08	0.29	−0.05	−0.07
Intimacy avoidance	−0.09	**0.38**	0.00	0.20	0.03	0.18
Manipulativeness	−0.03	0.02	**0.62**	−0.01	0.01	0.11
Deceitfulness	0.12	0.06	**0.70**	0.05	0.05	−0.02
Grandiosity	−0.03	0.04	**0.36**	*0.33*	0.12	0.06
Irresponsibility	−0.02	0.09	0.28	**0.16**	*-0.31*	0.27
Impulsivity	0.45	0.00	0.20	**0.17**	−0.05	0.09
Distractibility	0.24	0.19	0.11	**0.20**	−0.19	0.25
Perfectionism	0.08	0.04	0.08	0.03	**0.71**	0.08
Rigidity	0.04	0.11	0.05	−0.02	**0.59**	0.03
Orderliness	0.00	0.06	−0.07	*0.31*	**0.48**	0.17
Unusual beliefs and experiences	0.20	0.05	0.10	−0.08	0.07	**0.58**
Eccentricity	0.03	0.05	0.10	0.04	0.14	**0.68**
Perceptual dysregulation	0.04	0.07	−0.01	0.26	0.02	**0.45**

*Bolded are expected primary loadings of the facets [all of their loadings significant at p < 0.001 with the exception of Irresponsbility (p = 0.053), Impulsivity (p = 0.004), and Distractibility (p = 0.020)]. Italicized are non-primary loadings > |0.30|*.

* *For the sake of consistency, we used the original PID5BF+M label for this domain*.

Given that the six-factor solution of the independent version was less stable due to the lack of substantial facet loadings for the Disinhibition domain, we decided to explore this issue further by estimating exploratory factor analyses with varying number of factors (from one to eight) to see whether a different factor solution would provide a clearer pattern. The solutions with fewer than five factors showed poor model fit, suggesting that such factor structures did not accurately represent the data. The five-factor solution provided a clearer factor solution with regards to facet loadings then the six-factor version for the five PID domains sans Disinhibition while the facets of the Disinhibition domain loaded on other domains (impulsivity and withdrawal on Negative affectivity, irresponsibility on Antagonism). The seven- and eight-factor solutions provided an incremental improvement in model fit; however, they have not provided a clearer factor solution with regards to the pattern of facet loadings then the five- or six-factor solutions. The loading patterns for the five-, seven-, and eight-factor solutions and their model fit indices are provided in Appendixes 1–3.

Furthermore, we estimated a five-domain model in accordance with the five domains qualifiers defined in ICD-11. The fit of the model was good, $\chi_{\left(40\right)}^{2}$ = 162.41, *p* < 0.001, CFI = 0.961, RMSEA = 0.054, 90% RMSEA CI [0.046, 0.063]. The standardized loadings are shown in Table 3. The pattern of the loadings is similar to the six-factor PIDBF+M, again showing low (and not statistically significant) loadings for the facets of the Disinhibition domain, with separation insecurity showing high primary loading on this domain (λ = 0.78), with a non-significant loading on its primary facet (λ = 0.08).

**Table 3:** Loadings patterns of a 5-factor ICD-11 structure derived from the independent version of PID5BF+M.

	**Negative affectivity**	**Detachment**	**Antagonism**	**Disinhibition**	**Anankastia**
Emotional lability	**0.72**	−0.04	−0.04	0.12	0.10
Anxiousness	**0.35**	*0.32*	−0.11	0.27	0.07
Separation insecurity	**0.08**	−0.09	−0.01	*0.78*	−0.01
Withdrawal	0.09	**0.64**	0.04	−0.16	0.13
Anhedonia	−0.10	**0.52**	0.05	0.21	−0.05
Intimacy avoidance	−0.01	**0.47**	0.07	0.07	0.08
Manipulativeness	0.00	0.01	**0.66**	0.02	0.05
Deceitfulness	0.14	0.03	**0.62**	0.08	0.07
Grandiosity	−0.04	0.10	**0.38**	*0.33*	0.14
Irresponsibility	0.10	0.18	0.40	**0.07**	−0.24
Impulsivity	*0.54*	−0.01	0.21	**0.14**	0.00
Distractibility	*0.37*	0.26	0.20	**0.09**	−0.12
Perfectionism	0.07	0.00	0.06	0.04	**0.77**
Rigidity	0.04	0.07	0.02	−0.01	**0.60**
Orderliness	0.05	0.15	0.02	0.20	**0.50**

*Bolded are expected primary loadings of the facets [all of their loadings significant at p < 0.001 with the exception of Separation insecurity (p = 0.245), Irresponsibility (p = 0.339), Impulsivity (p = 0.017), and Distractibility (p = 0.143)]. Italicized are non-primary loadings > |0.30|*.

### Reliability

Internal reliabilities of the PID5BF+M scales in both samples are shown in Table 4. In the case of the PID5BF+M extracted from the 220-item version of the PID-5, the internal consistency was generally adequate, apart from low correlations for intimacy avoidance, irresponsibility, impulsivity, and rigidity. All domain reliabilities were satisfactory, with the average domain trait scores reliability of 0.71. The reliability of domains was as follows: Negative affectivity (ω = 0.72), Detachment (ω = 0.66), Antagonism (ω = 0.74), Disinhibition (ω = 0.73), Anankastia (ω = 0.69), and Psychoticism (ω = 0.74).

**Table 4:** Internal reliabilities of PID-5 facets and domains across two versions of PID5BF+M.

**Domain**	**Facet**	**Reliability 220-item version**		**Reliability 36-item version**	
Negative affectivity	Emotional lability	0.59	0.72	0.54	0.67
	Anxiousness	0.86		0.78	
	Separation insecurity	0.57		0.49	
Detachment	Withdrawal	0.60	0.66	0.51	0.59
	Anhedonia	0.51		0.54	
	Intimacy avoidance	0.44		0.57	
Antagonism	Manipulativeness	0.71	0.74	0.68	0.70
	Deceitfulness	0.64		0.49	
	Grandiosity	0.54		0.32	
Disinhibition	Irresponsibility	0.49	0.73	0.40	0.65
	Impulsivity	0.48		0.47	
	Distractibility	0.53		0.60	
Anankastia	Perfectionism	0.58	0.69	0.50	0.72
	Rigidity	0.49		0.57	
	Orderliness	0.58		0.64	
Psychoticism	Unusual beliefs and experiences	0.54	0.74	0.49	0.75
	Eccentricity	0.68		0.54	
	Perceptual dysregulation	0.58		0.53	
	Average reliability	0.58	0.71	0.54	0.68

*Reliability indexed by McDonald's ω*.

For the independent 36-item version, lower correlations were found for more facets than in the extracted version; these included separation insecurity, deceitfulness, grandiosity, irresponsibility, impulsivity, and unusual beliefs and experiences. In the case of the independent 36-item version of PID5BF+M, all domain reliabilities were satisfactory. The reliability of domains was as follows: Negative affectivity (ω = 0.67), Detachment (ω = 0.59), Antagonism (ω = 0.70), Disinhibition (ω = 0.65), Anankastia (ω = 0.72), and Psychoticism (ω = 0.75), with the average domain trait scores reliability of 0.68.

The results also showed good test-retest reliability of the underlying domains of the independent 36-item PID5BF+M: Negative affectivity *r* = 0.83, Detachment *r* = 0.73, Antagonism *r* = 0.79, Disinhibition *r* = 0.71, Anankastia *r* = 0.77, Psychoticism *r* = 0.81 (all *p* < 0.001). The test-retest correlations of facets ranged from *r* = 0.50 for irresponsibility to *r* = 0.82 for manipulativeness (all *p* < 0.001). Bivariate correlations for facets and domains across the two timepoints can be found in Appendix 4.

### Validity

#### Convergent Validity

The correlations between the facets derived from the original 220-item PID-5 and the facets defined by PID5BF+M are shown in Table 5. The correlations were high, ranging from *r* = 0.70 to *r* = 0.94, with an average of *r* = 0.81. The correlations between the five domains of the PID-5 and the extracted version of the PID5BF+M were also high, ranging from *r* = 0.86 to *r* = 0.93, with an average of *r* = 0.90.

**Table 5:** Correlations of selected facets and domains between the 220-item and the 36-item PID-5.

		**220-item version**																			
		**EMO**	**ANX**	**SEP**	**WIT**	**ANH**	**INT**	**MAN**	**DEC**	**GRAN**	**IRR**	**IMP**	**DIST**	**UNU**	**ECC**	**PCD**	**NA**	**DE**	**AN**	**DI**	**PS**
36-item version	EMO Emotional lability	0.77	0.58	0.39	0.25	0.22	0.06	0.25	0.33	0.23	0.29	0.46	0.48	0.33	0.45	0.54	0.71	0.23	0.31	0.50	0.50
	ANX Anxiousness	0.59	0.88	0.41	0.38	0.40	0.11	0.21	0.33	0.24	0.22	0.31	0.52	0.30	0.46	0.52	0.76	0.37	0.30	0.44	0.49
	SEP Separation insecurity	0.43	0.47	0.82	0.17	0.35	0.11	0.22	0.27	0.19	0.29	0.36	0.39	0.24	0.34	0.38	0.69	0.25	0.26	0.42	0.36
	WIT Withdrawal	0.26	0.44	0.07	0.81	0.45	0.33	0.24	0.31	0.23	0.23	0.17	0.38	0.21	0.39	0.34	0.31	0.69	0.31	0.32	0.37
	ANH Anhedonia	0.36	0.44	0.30	0.51	0.75	0.26	0.25	0.36	0.21	0.36	0.36	0.53	0.26	0.38	0.43	0.45	0.63	0.32	0.51	0.41
	INT Intimacy avoidance	0.18	0.23	0.12	0.42	0.34	0.78	0.22	0.26	0.24	0.23	0.18	0.28	0.22	0.29	0.35	0.21	0.65	0.28	0.28	0.32
	MAN Manipulativeness	0.24	0.27	0.16	0.34	0.25	0.17	0.82	0.75	0.49	0.48	0.29	0.31	0.39	0.43	0.41	0.27	0.33	0.80	0.41	0.47
	DEC Deceitfulness	0.36	0.37	0.31	0.32	0.26	0.14	0.63	0.81	0.43	0.51	0.37	0.44	0.38	0.44	0.45	0.42	0.31	0.73	0.52	0.48
	GRAN Grandiosity	0.30	0.37	0.23	0.32	0.28	0.21	0.44	0.49	0.79	0.37	0.27	0.31	0.40	0.41	0.40	0.36	0.34	0.67	0.37	0.46
	IRR Irresponsibility	0.41	0.34	0.25	0.35	0.28	0.18	0.42	0.52	0.39	0.76	0.53	0.57	0.42	0.56	0.52	0.41	0.35	0.52	0.72	0.58
	IMP Impulsivity	0.53	0.40	0.39	0.27	0.32	0.10	0.31	0.42	0.27	0.47	0.94	0.55	0.30	0.46	0.49	0.54	0.29	0.39	0.80	0.48
	DIST Distractibility	0.54	0.54	0.28	0.39	0.35	0.11	0.23	0.36	0.20	0.45	0.50	0.84	0.29	0.55	0.54	0.55	0.36	0.31	0.73	0.54
	UNU Unusual Beliefs and experiences	0.39	0.40	0.21	0.28	0.23	0.18	0.40	0.44	0.38	0.38	0.30	0.40	0.80	0.61	0.60	0.41	0.30	0.48	0.43	0.76
	ECC Eccentricity	0.43	0.44	0.25	0.38	0.27	0.17	0.38	0.45	0.41	0.47	0.39	0.49	0.49	0.87	0.60	0.45	0.35	0.48	0.53	0.78
	PCD Perceptual dysregulation	0.34	0.34	0.23	0.32	0.32	0.30	0.30	0.37	0.33	0.37	0.39	0.39	0.49	0.47	0.70	0.37	0.39	0.39	0.46	0.61
	NA Negative affectivity	0.75	0.81	0.67	0.33	0.40	0.11	0.29	0.39	0.28	0.34	0.48	0.58	0.37	0.52	0.60	0.91	0.35	0.37	0.57	0.57
	DE Detachment	0.35	0.49	0.21	0.77	0.67	0.58	0.31	0.40	0.30	0.36	0.31	0.52	0.30	0.47	0.48	0.43	0.86	0.39	0.48	0.48
	AN Antagonism	0.37	0.41	0.30	0.40	0.32	0.21	0.77	0.85	0.69	0.56	0.39	0.45	0.47	0.53	0.51	0.44	0.39	0.90	0.55	0.58
	DI Disinhibition	0.62	0.53	0.38	0.41	0.39	0.15	0.38	0.53	0.35	0.67	0.82	0.81	0.41	0.64	0.64	0.62	0.40	0.49	0.93	0.65
	PS Psychoticism	0.48	0.50	0.29	0.41	0.34	0.26	0.46	0.52	0.47	0.52	0.44	0.53	0.75	0.83	0.78	0.51	0.42	0.57	0.59	0.91

*Coefficients with r > 0.30 are marked in gray with an increase in darkness reflecting the strength of the association. All coefficients > 0.10 significant at p < 0.05, coefficients > 0.11 significant at p < 0.01, coefficients > 0.12 significant at p < 0.001*.

In order to test the similarity of both versions of PID5BF+M, the Sample 1 with the extracted version and the Sample 2 with the independent 36-item version were combined in a multigroup model. The fit of the configural model was $\chi_{\left(120\right)}^{2}$ = 255.89, *p* < 0.001, CFI = 0.979, RMSEA = 0.037, 90% RMSEA CI [0.031, 0.043], while the fit of the most constrained scalar model was $\chi_{\left(204\right)}^{2}$ = 438.18, *p* < 0.001, CFI = 0.965, RMSEA = 0.037, 90% RMSEA CI [0.032, 0.042]. The difference between the models was S-B Δ$\chi_{\left(84\right)}^{2}$ = 182.02, *p* < 0.001, ΔCFI = −0.015, ΔRMSEA = < -0.0001. Thus, there was partial evidence that the fit of the PID5BF+M extracted from the full 220-item PID-5 and its independent 36-item version was not dissimilar.

#### Predictive Validity

We tested the predictive validity of the independent 36-item PID5BF+M to determine borderline pathology according to ICD-11 by assessing associations with the IPDEQ criteria for emotionally unstable PDs of both types (i.e., borderline, and impulsive). The results showed that the highest positive correlation for the borderline personality subdomain was found with Negative affectivity (*r* = 0.51), while for the impulsive subdomain it was with Disinhibition (*r* = 0.47, both *p* < 0.001). The correlations were lower for other domains (e.g., correlation of the borderline type with Anankastia *r* = 0.12, correlation of the impulsive type with Anankastia *r* = 0.11, both *p* < 0.001). In line with the definition of the ICD-11 Borderline Pattern qualifier, the highest positive correlations for the emotionally unstable PD as such were found with Negative affectivity (*r* = 0.55) and Disinhibition (*r* = 0.53, both *p* < 0.001). These associations are shown in Table 6.

**Table 6:** Correlations of 36-item PID5BF+M domains and IPDE-defined emotionally unstable PD.

	**IPDE BP**	**IPDE IMP**	**IPDE combined**
Negative affectivity	0.51	0.42	0.55
Detachment	0.35	0.20	0.33
Antagonism	0.32	0.39	0.42
Disinhibition	0.42	0.47	0.53
Ananakastia	0.12	0.11	0.14
Psychoticism	0.37	0.36	0.43

*BP, borderline subtype; IMP, impulsive subtype. All correlations significant at p < 0.001*.

## Discussion

The current study aimed to assess the validity of the shortened version of PID-5, the PIDBF+M, using two non-clinical Czech samples. The validity of the measure was assessed in two forms: first, as extracted from the original 220-item PID-5; second, when the PIDBF+M was administered as a stand-alone measure. Evaluation of psychometric properties of the independent PID5BF+M seems to be an important step toward disseminating the dimensional diagnostic approach to a broad range of clinicians who, with international adaptations of ICD-11, urgently need short but reliable instruments for PD diagnostics within the new system (16).

### Factor Structure and the Model Fit

In terms of maintaining continuity with the previous research (11, 16), our first goal in the current study was the validation of the factor structure of PID5BF+M, extracted from the original 220-item PID-5. The presented 6-factor model, combining the ICD-11 and DSM-5 domains of personality traits (i.e., Negative affectivity, Detachment, Antagonism, Disinhibition, Anankastia, and Psychoticism), was validated using a sample of respondents from the general population. A good model fit for the 6-factor ESEM model suggested that the proposed structure of three facets per domain fit our data well. This confirms findings from the previous study (11) and further demonstrates the utility of employing the shorter version of PID-5 instead of the full one.

However, we noted some problems with the Disinhibition domain that deserve a more detailed comment. First, there was the issue of irresponsibility facet loading primarily on Psychoticism and only secondarily on Disinhibition, its respective domain. Although there was a relatively small difference between loadings found in this study (λ = 0.37 vs. λ = 0.36), it can be seen as further evidence of proneness of the irresponsibility factor to cross-loadings, previously demonstrated in the extracted PID5BF+M by other studies using the original 220-item version of PID-5 [e.g., (31, 32)]. It should be noted that these studies mostly employed samples from the general population or mixed samples with a predominance of respondents from the general population (11). Since PID-5 is a tool primarily intended for the evaluation of personality psychopathology (33), there is a presumption of the greater stability of individual factors within the clinical population. From the perspective of common clinical practice, a primary loading of the separation insecurity facet on Disinhibition instead of Negative affectivity also seems justified. Since in this case, too, the difference between the primary and secondary loadings was only minor (λ = 0.33 vs. λ = 0.31), it can be interpreted in the context of the close interconnection between the two domains in relation to borderline psychopathology in AMPD (34), as well as in the traditional descriptive concept of borderline PD according to DSM-5 Section II (3, 11) and also of the emotionally unstable PD according to ICD-10 (35).

Despite the fact that the separation insecurity facet from the extracted PID5BF+M showed a substantial primary loading on the Negative affectivity domain across international samples (11), including the mixed Czech sample (8), the problematic primary loading of this facet in the current study was even more pronounced in the case of the independent PID5BF+M. While a weakening of the separation insecurity factor loading on Negative affectivity can be attributed to the reduction of items in the independent version of the measure, the large size of its factor loading on Disinhibition domain is rather unexpected given the current knowledge about the internal structure of PID-5 (18). It is possible that this finding stems from different perception of the relevant items in both Czech samples due to translation and, as such, might be idiosyncratic to the current samples and not constitute a meaningful factor on its own.

The proposed 6-factor structure of PID5BF+M was also replicated in the 36-item independent version. Nevertheless, the issue of problematic loadings of the three primary facets of the Disinhibition domain was even more prominent in this version. Although our results confirmed the existence of negative cross-loading of the irresponsibility facet on the Anankastia domain and the cross-loading of distractibility on the Negative affectivity and Psychoticism domains (11), the Disinhibition domain virtually disintegrated in the independent version. This fact is evidenced, among other things, by the primary loading of the impulsivity facet on the Negative affectivity and only the tertiary loading on Disinhibition. Although this is an issue previously discussed in the context of personality trait models in both DSM-5 (18) and ICD-11 (36), the question is to what extent the disintegration of the Disinhibition domain might be ascribed to the shorter version of the inventory where each facet is defined only by two items. One can hypothesize that the context in which the selected items are presented might affect the respondent. The randomized order of items in the original version of PID-5 (5) can, due to its length, strengthen the respondent's ability to differentiate between the latent meanings of individual statements. However, this hypothesis could not be verified in this study because the independent version of PID5BF+M was administered to a different sample of respondents than the version extracted from the original PID-5. Nevertheless, the definition of the Disinhibition domain in the current study via primary facet loading of separation insecurity and other substantial facet loadings of anhedonia, grandiosity, anxiousness and orderliness is of particular clinical interest, as it is somewhat reminiscent of the thin-skinned narcissism (37). According to Bateman (38), such patients experience discomfort, shame, and anxiety when feeling rejected. In response, they seek complete agreement with the object, thereby denying all differences between themselves and the other. However, due to the differences in factor loadings between the samples in the Disinhibition domain, these conclusions need to be handled with caution, as they deserve more thorough evaluation in the clinical population.

The loading pattern in the 5-factor ICD-11 version was very similar to the original, six-factor version of the PID5BF+M, suggesting that the above-mentioned issues with low loadings especially for the Disinhibition domain were not alleviated by the omission of the Psychoticism domain.

### Reliability

Although the obtained values of internal consistency are significantly lower in comparison with the original version of PID-5 for both versions of PID-5BF+M, they were still largely satisfactory, both at the level of trait domains and the majority of individual trait facets. This indicates good reliability of the measure despite the substantial reduction in the number of items compared to the previously performed studies [e.g., (5, 39, 40)]. On the domain level, the only exception is Detachment, which showed the lowest reliability in both samples. This result is quite surprising, as this domain generally achieves good internal consistency values in the original version of PID-5 [e.g., (41, 42)] and its shortened versions PID-5-BF (43) and PID5BF+ (16). In addition, McDonald's omega should be a less ambiguous indicator of internal consistency than Cronbach's alpha, which is, to a large extent, a function of the number of test items and the mean of inter-item correlation (44–47). Compared to PID5BF+, however, PID5BF+M in both our samples demonstrated satisfactory internal consistency of the Anankastia domain, which provides support for solving the issue of the rigid perfectionism facet and the perseveration facet overlap (48) by dividing rigid perfectionism into rigidity and perfectionism, and adding a separate facet of orderliness within PID5BF+M (11).

Regarding the stability of the independent version of PID5BF+M over time, although the test-retest period in our study varied (ranging from 1 to 15 weeks), the average test-retest interval of ~1.5 months seems to be sufficient compared to other studies [e.g., (21, 44, 49)] when we consider the total number of participants in the retest (*n* = 201). The values of the coefficient *r* indicate good consistency of scores for all domains and facets of the independent version PID5BF+M over an average period of 1.5 months. These results can be considered as probably the first confirmation of PID5BF+M as a stand-alone measure in terms of temporal stability and occasional specificity.

### Convergent Validity

In assessing the convergent validity of PID5BF+M, we compared the facets and domains of the 36-item version of the questionnaire with the 220-item PID-5 in this study. Because we administered the full version of PID-5 only to Sample 1, an independent version of PID5BF+M could not be included in these analyses. Another issue was the Anankastia domain, which is defined in PID5BF+M by a modified triplet of facets-namely by rigidity, perfectionism and orderliness-unlike PID5BF+, where Anankastia is defined by the facets of rigid perfectionism and perseveration, i.e., facets included in the original version of the PID-5. Given the fact that Anankastia is an integral part of the personality trait model in ICD-11, a more thorough assessment of the convergent and predictive validity of this domain would be appropriate. Nevertheless, the average correlations of 0.81 at the facet level of five of the original PID-5 domains (i.e., Negative affectivity, Disinhibition, Detachment, Antagonism, and Psychoticism) and 0.90 at the domain level confirmed good convergent validity in line with previous studies with PID5BF+ (16) and PID-5 (50).

In terms of using PID5BF+M as an independent 36-item measure in a routine clinical practice, an important step was to verify the potential differences between the independent and the extracted version of the measure. In our study, the two models were largely invariant, as we found minimal differences in terms of model fit between the configural and the scalar model (all loadings and intercepts constrained to be equal across the samples). However, given that the two samples came from different populations, we urge caution in interpreting this finding as a definite proof of the invariance of the two measures (see Limitations for further discussion of this issue). What our study indicates is that the 220-item version and the 36-item version of PID5BF+M, each estimated in a specific sample, were not dissimilar in terms of factor structure, item loadings, and item intercepts, at least based on change in relative model fit indices.

These results support the consideration of an independent version of the 36-item PID5BF+M as a valid diagnostic tool for assessing maladaptive personality traits according to both DSM-5 and ICD-11. Although this conclusion is consistent with the clinicians' demand for short and valid tools that minimize the patient burden while providing reliable data, it should be borne in mind that the 220-item PID-5 was designed to achieve the most detailed description of patient's strengths and weaknesses (33). The potentially negative effect of item reduction on the validity of PID-5 has also been discussed in the case of PID-5-BF (44). Psychodynamically oriented authors perceive the operationalization of the model of personality traits in ICD-11 and AMPD based on self-assessment as a procedure more suitable for research rather than for daily clinical practice (51). This somewhat contrasts with the predominance of self-assessment tools that have emerged since the publication of PID-5 in 2012 (52). Based on our findings, having in mind the problematic factor loading of the Disinhibition domain in case of the independent version, we recommend perceiving the independent 36-item PID5BF+M as a screening tool, whose results can inform the clinician's decision regarding the administration of other diagnostic methods.

### Predictive Validity

The main goal of introducing the Borderline Pattern qualifier within the ICD-11 model for PDs, which virtually reflects the diagnostic criteria for the borderline PD in DSM-5 section II, was to maintain diagnostic continuity between the categorical and dimensional models while ensuring the smallest possible overlap of borderline PD with other PDs (53, 54). Although the inclusion of this qualifier can be considered a controversial step considering the purely dimensional ICD-11 model (14), it becomes meaningful when assessing the evidence for a psychotherapeutic effect on the treatment of borderline PD. The ability of the independent version of PID5BF+M to predict emotionally unstable psychopathology defined by the ICD-10 criteria was confirmed in our study via strong correlations in the expected direction of Negative affectivity and Disinhibition domains. These domains are primary domains for defining borderline PD based on both, the ICD-11 personality trait qualifiers (11, 16, 20) and IPDEQ self-assessment. To some extent, the tertiary correlation of both types of emotionally unstable PD (i.e., borderline and impulsive) on the Psychoticism domain confirms the clinical experience with the quasi-psychotic phenomena and transient dissociative states in this group of patients (55, 56).

### Limits and Future Directions

The results of our study contribute to the current state of knowledge in several ways. First, we have confirmed the independent 36-item PID5BF+M as a reliable and valid tool to generally assess personality psychopathology, in accordance with the proposed dimensional model of maladaptive personality traits according to ICD-11, as well as DSM-5 AMPD. We have also demonstrated the predictive validity of this measure in relation to the assessment of borderline psychopathology in line with the transition from categorical to a dimensional diagnosis of PDs. Nevertheless, our findings need to be considered with respect to certain limitations that may inspire future research. We consider the absence of a clinical group of patients to be the main limitation of this study. The inclusion of a clinical cohort could have helped answer the question of the problematic factor loading of the Disinhibition domain. In addition, the disintegration of this domain in the case of the PID5BF+M independent version makes it impossible to establish normative values. Relatedly, instead of “Disinhibition,” the pattern of loadings for this domain in the 36-item extracted version could have warranted using a different label. However, it is not clear whether this finding truly reflects a different factor structure or whether this finding is idiosyncratic to the current data. In this respect, the independent version of the tool should be further examined in international samples to verify the possible impact of translation or cultural specificity. It is also necessary to point out that Sample 1 and Sample 2 differed in terms of sex ratio, average age, or highest attained education, as they were convenience samples drafted from different populations. As such, we cannot rule out the possibility that the lack of substantial differences between the independent and the extracted version of PID5BF+M might be due to differences in the characteristics of these samples. However, past research on the PID5BF+M does not suggest that these characteristics would substantially affect the structure of the model (11). Although some authors point to the possible effect of validity bias due to gender imbalance (16), in our view, the essential invariance found when comparing these two heterogeneous samples suggests that the PID5BF+M model might be robust to the effect of the demographic variables. From the point of view of convergent validity, another limitation is the restricted possibility to verify the validity of the newly defined Anankastia domain in PID5BF+M, which is not part of the original version of PID-5. In this regard, we consider it appropriate to explore the convergence with other tools for the ICD-11 trait model, such as Personality Inventory for ICD-11 (PiCD) (9). From the point of view of predictive validity, the exclusive use of the IPDEQ scale for emotionally unstable PD can be considered another limitation of our study. Although the selected items correspond relatively accurately to the criteria of the ICD-11 Borderline Pattern qualifier, their limited number (i.e., one item = one criterion) may reduce its predictive power to some extent. Future research could therefore use one of the more comprehensive tools for assessing the borderline PD based on self-assessment, such as the Borderline Personality Questionnaire (BPQ) (57). Finally, a broad range of the test-retest interval might be another limitation of the study.

## Data Availability Statement

The raw data supporting the conclusions of this article will be made available by the authors, without undue reservation.

## Ethics Statement

The studies involving human participants were reviewed and approved by Ethics Committee of the General University Hospital in Prague Na Bojišti 1, 128 08 Prague 2. The patients/participants provided their written informed consent to participate in this study.

## Author Contributions

KR drafted this paper and was responsible for its final version. AK conducted all data analyses. LS was responsible for data collection. All authors contributed to the article and approved the submitted version.

## Conflict of Interest

The authors declare that the research was conducted in the absence of any commercial or financial relationships that could be construed as a potential conflict of interest. The handling editor declared a past co-authorship with one of the authors KR.

## Footnotes

^1^In the remaining part of the manuscript, we employed the label “Disinhibition” and used its original facets (i.e., impulsivity, irresponsibility, distractibility) to compute this domain score in the subsequent analyses. This decision was made for the purpose of consistency and comparison. However, we acknowledge that this domain was not well-defined by its respective three facets in Sample 2 (see more in Discussion).
