# Supplementary material for: Psychometric Properties of the Independent 36-Item PID5BF+M for ICD-11 in the Czech-Speaking Community Sample
Source: Front Psychiatry. 2021 May 26;12:643270. doi: 10.3389/fpsyt.2021.643270 (PMC8187568; doi:10.3389/fpsyt.2021.643270)
Supplement: Supplementary file 1 [file Data_Sheet_1.docx]

| Appendix 1 | | | | | |
| --- | --- | --- | --- | --- | --- |
|  | | | | | |
| *Loading patterns of the five-factor solution for the independent version of PID5BF+M* | | | | | |
|  | | | | | |
|  | Negative Affectivity | Detachment | Antagonism | Anankastia | Psychoticism |
| Emotional lability | **.71** | -.06 | .01 | .10 | .11 |
| Anxiousness | **.48** | *.35* | -.03 | .10 | .00 |
| Separation insecurity | **.46** | .07 | .15 | .07 | -.05 |
| Withdrawal | -.04 | **.52** | .02 | .11 | .08 |
| Anhedonia | .00 | **.60** | .11 | -.02 | -.08 |
| Intimacy avoidance | -.05 | **.47** | .02 | .05 | .18 |
| Manipulativeness | -.07 | .00 | **.65** | .01 | .11 |
| Deceitfulness | .11 | .04 | **.69** | .06 | .00 |
| Grandiosity | .10 | .16 | **.41** | .14 | .06 |
| Irresponsibility | .03 | .15 | *.32* | -.29 | .27 |
| Impulsivity | *.53* | .00 | .22 | -.02 | .12 |
| Distractibility | *.32* | .23 | .13 | -.16 | .27 |
| Perfectionism | .05 | .00 | .08 | **.74** | .08 |
| Rigidity | .00 | .05 | .04 | **.60** | .04 |
| Orderliness | .11 | .18 | -.02 | **.49** | .16 |
| Unusual beliefs and exp. | .11 | -.02 | .08 | .08 | **.61** |
| Eccentricity | -.01 | .05 | .09 | .14 | **.70** |
| Perceptual dysregulation | .14 | .16 | .03 | .04 | **.44** |
| *Note.* Bolded are expected primary loadings of the facets (all of their loadings significant at *p* <.001). Italicized are non-primary loadings > \|.30\|.  Model fit: χ2(73) = 308.58, *p* <.001, CFI = .943, RMSEA = .056, 90% RMSEA CI [.049, .062]. | | | | | |

| Appendix 2 |  |  |  |  |  |  |  |  |
| --- | --- | --- | --- | --- | --- | --- | --- | --- |
|  |  |  |  |  |  |  |  |  |
| *Loading patterns of the seven-factor solution for the independent version of PID5BF+M* | | | | | | | |  |
|  |  |  |  |  |  |  |  |  |
|  | Negative affectivity | Detachment | Antagonism | Separation Insecurity | Disinhibition | Anankastia | Psychoticism |  |
| Emotional lability | **.43** | -.08 | .02 | -.10 | .43 | .09 | .13 |  |
| Anxiousness | **.72** | .17 | .01 | .08 | .01 | -.01 | .03 |  |
| Separation insecurity | *.33* | -.24 | .08 | **.40** | .17 | .03 | -.08 |  |
| Withdrawal | .13 | **.68** | .06 | -.09 | .00 | .09 | .04 |  |
| Anhedonia | .15 | **.39** | .10 | .27 | -.06 | -.05 | -.11 |  |
| Intimacy avoidance | -.08 | **.42** | -.06 | .28 | .11 | .06 | .08 |  |
| Manipulativeness | -.01 | .02 | **.65** | .04 | -.02 | -.01 | .12 |  |
| Deceitfulness | .07 | .05 | **.65** | .01 | .16 | .05 | .00 |  |
| Grandiosity | .05 | .00 | **.34** | *.34* | .04 | .12 | .02 |  |
| Irresponsibility | -.13 | .13 | .22 | .24 | **.19** | -.27 | .18 |  |
| Impulsivity | .02 | .01 | .06 | .06 | **.79** | .03 | -.02 |  |
| Distractibility | .13 | .17 | .06 | .16 | **.31** | -.15 | .19 |  |
| Perfectionism | .04 | .03 | .07 | .04 | .05 | **.72** | .08 |  |
| Rigidity | .01 | .11 | .03 | -.02 | .07 | **.61** | .03 |  |
| Orderliness | .15 | .03 | -.06 | *.31* | -.02 | **.46** | .13 |  |
| Unusual beliefs and exp. | .13 | .02 | .11 | -.06 | .06 | .03 | **.65** |  |
| Eccentricity | .00 | .07 | .09 | .13 | .04 | .12 | **.63** |  |
| Perceptual dysregulation | .07 | .05 | -.02 | .29 | .09 | .01 | **.40** |  |
| *Note.* The domain names for factors that do not align with the original PID-5BF+M domains are suggestions. Bolded are the largest loadings of each facet (all of their loadings significant at *p* <.001 with the exception of anhedonia *p* <.027, separation insecurity *p* = .257). Italicized are non-primary loadings > \|.30\|.  Model fit: χ2(48) = 92.01, *p* <.001, CFI = .989, RMSEA = .030, 90% RMSEA CI [.020, .039]. | | | | | | | | |

| Appendix 3 |  |  | |  | |  | |  | |  | |  | | |  |
| --- | --- | --- | --- | --- | --- | --- | --- | --- | --- | --- | --- | --- | --- | --- | --- |
|  |  |  | |  | |  | |  | |  | |  | | |  |
| *Loading patterns of the eight-factor solution for the independent version of PID5BF+M* | | | | | | | | | | | | | | | |
|  |  |  | |  | |  | |  | |  | |  | | |  |
|  | Anxiousness | | Separation insecurity | | Detachment | | Antagonism | | Anankastia | | Emotional instability | | Unusual beliefs and exp. | Psychoticism | |
| Emotional lability | *.31* | | .10 | | -.11 | | .00 | | .10 | | **.46** | | .13 | -.03 | |
| Anxiousness | **.80** | | .10 | | .07 | | .02 | | -.02 | | .02 | | .03 | .02 | |
| Separation insecurity | .11 | | **.71** | | -.04 | | -.02 | | .00 | | .09 | | .03 | -.02 | |
| Withdrawal | .20 | | -.18 | | **.56** | | .06 | | .10 | | .03 | | .08 | -.01 | |
| Anhedonia | .13 | | .17 | | **.50** | | .07 | | -.06 | | -.08 | | -.03 | .00 | |
| Intimacy avoidance | -.04 | | .07 | | **.48** | | -.06 | | .05 | | .07 | | .05 | .20 | |
| Manipulativeness | .01 | | .02 | | -.01 | | **.66** | | .00 | | -.02 | | .07 | .10 | |
| Deceitfulness | .07 | | .07 | | .05 | | **.63** | | .04 | | .16 | | .06 | -.03 | |
| Grandiosity | -.01 | | **.33** | | .13 | | *.31* | | .10 | | .00 | | .05 | .11 | |
| Irresponsibility | -.10 | | .06 | | .14 | | .23 | | -.28 | | .16 | | .04 | **.31** | |
| Impulsivity | .00 | | .13 | | .03 | | .06 | | .02 | | **.71** | | .03 | .04 | |
| Distractibility | .16 | | .01 | | .12 | | .08 | | -.15 | | .32 | | .03 | *.32* | |
| Perfectionism | .03 | | .06 | | .03 | | .05 | | **.72** | | .05 | | .08 | .05 | |
| Rigidity | .02 | | .00 | | .10 | | .03 | | **.61** | | .07 | | .05 | -.01 | |
| Orderliness | .19 | | .13 | | .00 | | -.03 | | **.46** | | -.05 | | -.03 | *.36* | |
| Unusual beliefs and exp. | .03 | | .02 | | .01 | | .02 | | -.01 | | .01 | | **.99** | .01 | |
| Eccentricity | .03 | | -.01 | | .03 | | .11 | | .14 | | .05 | | .32 | **.43** | |
| Perceptual dysregulation | .08 | | .11 | | .05 | | -.01 | | .01 | | .05 | | .19 | **.43** | |
| *Note.* The domain names for factors that do not align with the original PID-5BF+M domains are suggestions. Bolded are the largest loadings of each facet (all of their loadings significant at *p* <.001). Italicized are non-primary loadings > \|.30\|.  Model fit: χ2(37) = 44.91, *p* <.001, CFI = .98, RMSEA = .014, 90% RMSEA CI [.000, .027]. | | | | | | | | | | | | | | | |

| Appendix 4 |  |
| --- | --- |
|  |  |
| *Test-retest correlations for PIDBF+M facets and domains* | |
|  |  |
|  | test-retest correlation |
| Emotional lability | .80 |
| Anxiousness | .72 |
| Separation insecurity | .76 |
| Withdrawal | .65 |
| Anhedonia | .65 |
| Intimacy avoidance | .73 |
| Manipulativeness | .82 |
| Deceitfulness | .68 |
| Grandiosity | .54 |
| Irresponsibility | .50 |
| Impulsivity | .68 |
| Distractibility | .67 |
| Perfectionism | .72 |
| Rigidity | .69 |
| Orderliness | .58 |
| Unusual beliefs and experiences | .72 |
| Eccentricity | .77 |
| Perceptual dysregulation | .64 |
| Negative affectivity | .83 |
| Detachment | .73 |
| Antagonism | .79 |
| Disinhibition | .71 |
| Anankastia | .77 |
| Psychoticism | .81 |
| *Note*. All correlation estimates significant at *p* <.001. | |
